# Teachers’ dissatisfaction during the COVID-19 pandemic: Factors contributing to a desire to leave the profession

**DOI:** 10.3389/fpsyg.2022.940718

**Published:** 2022-09-14

**Authors:** Amreen Gillani, Rhodri Dierst-Davies, Sarah Lee, Leah Robin, Jingjing Li, Rebecca Glover-Kudon, Kayilan Baker, Alaina Whitton

**Affiliations:** ^1^Deloitte Consulting LLP, Atlanta, GA, United States; ^2^Division of Population Health, National Center for Chronic Disease Prevention and Health Promotion, Centers for Disease Control and Prevention, Atlanta, GA, United States; ^3^Division of Adolescent and School Health, National Center for HIV, Hepatitis, STD, and Tuberculosis Prevention, Centers for Disease Control and Prevention, Atlanta, GA, United States; ^4^Division of Overdose Prevention, National Center for Injury Prevention and Control, Centers for Disease Control and Prevention, Atlanta, GA, United States; ^5^Division of Non-Infectious Diseases, National Center on Birth Defects and Developmental Disabilities, Centers for Disease Control and Prevention, Atlanta, GA, United States; ^6^National Foundation for the Centers for Disease Control and Prevention (CDC Foundation), Atlanta, GA, United States

**Keywords:** teachers, job satisfaction, retention, mental health, school policies, prevention strategies, COVID-19

## Abstract

**Introduction:**

The COVID-19 pandemic required more responsibilities from teachers, including implementing prevention strategies, changes in school policies, and managing their own mental health, which yielded higher dissatisfaction in the field.

**Methods:**

A cross-sectional web survey was conducted among educators to collect information on their experiences teaching during the COVID-19 pandemic throughout the 2020–2021 academic year. Qualtrics, an online survey platform, fielded the survey from May 6 to June 8, 2021 to a national, convenience sample of 1,807 respondents.

**Results:**

Findings revealed that overall, 43% of K-12 teachers reported a greater intention to leave the profession than previously recalled prior to the COVID-19 pandemic. Intention to leave was multi-level, and associated with socio-demographic factors (e.g., age: AOR = 1.87, *p* < 0.05), individual factors (e.g., perceived COVID risks: AOR = 1.44, *p* < 0.05), and teachers’ agency (e.g., dissatisfaction with school/district communications and decisions: AOR = 1.34, *p* < 0.05). We also found demographic disparities with respect to race and gender (e.g., female teachers: AOR: 1.78, *p* < 0.05) around teachers’ ability to provide feedback to schools on opening/closing and overall dissatisfaction with school/district COVID-19 prevention strategies implementation and policies.

**Conclusion:**

These findings are consistent with the Job-Demand and Resources Model (JD-R), which posits that lack of organizational support can exacerbate job stressors, leading to burnout. Specifically, dissatisfaction with the way school policies were implemented took a toll on teachers’ mental health, leading to a desire to leave the profession. These findings are also consistent with research conducted once in-person teaching resumed in 2020–2021, specifically that the COVID-19 pandemic exacerbated preexisting teacher shortages that led to self-reported issues of stress, burnout, and retention.

**Implications:**

Further research is necessary to understand the resources that may be most useful to reduce the demands of teaching in the context of the COVID-19 pandemic. Some teachers are more likely to leave the field, and educational agencies may wish to target their teacher-retention efforts with emphasis on strong employee wellness programs that help educators to manage and reduce their stress. Education agency staff may wish to review policies and practices to provide meaningful opportunities to give input to school/district decisions and enable proactive communication channels.

## Introduction

Even before the onset of the COVID-19 pandemic, teaching was recognized as one of the most stressful professions in the United States, on par with physicians and service workers (Gallup, 2014). Stress among teachers is associated with negative physical and mental health outcomes, both of which are strongly correlated with burnout, low job satisfaction, and poor retention (García-Carmona et al., 2019; Diliberti et al., 2021; Farley and Chamberlain, 2021; Ozamiz-Etxebarria et al., 2021). Prior to the pandemic, the general mental health among teachers was declining nationally (Farley and Chamberlain, 2021). While we are only beginning to understand the additional impacts of COVID-19 on the teaching profession, initial indicators suggest that the pandemic poses an added mental health strain. Due to this and other challenges to the profession, the United States may be facing a teacher retention crisis at a time when there is already a labor shortfall in the education sector (U.S. Department of Labor, 2021).

Researchers have used multiple constructs and frameworks to contextualize issues of burnout and retention in workplace settings such as schools. Burnout, often referred to as burnout syndrome, is characterized as a complex condition associated with physical and emotional exhaustion, low self-efficacy, fatigue, depersonalization, and chronic stress (Weber and Jaekel-Reinhard, 2000; Kaschka et al., 2011; Chirico, 2016). Classic frameworks of occupational health, particularly the Job-Demand and Resources Model (JD-R), are instrumental to operationalize the associations among burnout, stress, and workplace attrition that many teachers are experiencing (Bakker et al., 2004). JD-R model posits that exhaustion in meeting work demands leads to emotional withdrawal (i.e., disengagement), while job resources that buffer job demands (i.e., autonomy, policies, latitude of decision-making) help alleviate it.

Historically, approximately 8% of teachers leave the profession annually, most voluntarily, due to reasons ranging from poor pay, lack of career advancement, and most notably stress (Podolsky et al., 2016; McFeely, 2018; Diliberti et al., 2021). Sources of stress among teachers are varied, including those unique to the profession such as overemphasis on academic testing, student behavioral problems, and other stressors such as lack of strong school leadership, increasing job demands, limited work resources, and lack of input and control over school policies. Additionally, teacher stress and burnout negatively impact students, leading to decreased quality of education and achievements. Taken together, identifying and understanding root causes of stress among teachers is necessary to support not only retention, but teachers’ health and sustainability of their careers.

The COVID-19 pandemic exacerbated these preexisting teacher shortages in the United States, and spotlighted issues of stress, burnout, and retention that have long plagued the profession (Carver-Thomas et al., 2021). In response to the pandemic, schools across the U.S. turned to virtual learning at the end of the 2019–2020 academic years. By the 2020–2021 school year, instruction was being accomplished by a mix of in-person, virtual instruction, or a hybrid approach. Additionally, adoption of prevention strategies such as physical distancing, masking, and cleaning and disinfecting, which were paramount to preventing exposure and spread, was mixed across the country. Often, teachers were tasked with adoption of new teaching models and enforcement of various prevention measures with tight timelines with little resources or input from school or district officials. A recently published article highlighted that for some teachers stress and anxiety increased within the first 2 months of the pandemic due to novel technology adoption (Pressley et al., 2021). They also bore the risk of workplace exposure to COVID-19, impacting both them personally and their loved ones at home, who may have conditions that elevated their risk of complications (Claxton et al., 2020).

One study found that 4 in 10 teachers who left their jobs cited challenges in teaching virtually or in a mix of in-person and virtual instruction as the primary reason (Diliberti et al., 2021). Another found that one in four teachers wanted to leave the profession by the end of the 2020–2021 academic year, compared to one in six prior to the pandemic (Steiner and Woo, 2021). A third study found that teachers who left during the pandemic reported stress as the most common reason (Diliberti et al., 2021). Additionally, a report examining teacher shortages in California found that some open positions were filled by less qualified staff than their predecessors, which could increase the gap in students’ learning loss and education inequality (Carver-Thomas et al., 2021).

Factors such as uncertainty and controversies regarding the COVID-19 pandemic’s progression, institutional policies on COVID-19 prevention strategies, and changing instructional expectations add more strains on classroom teachers (Ozamiz-Etxebarria et al., 2021). As COVID-19 continues to impact teachers, a deeper understanding of what interpersonal and school-based factors have on retention can be beneficial for supporting teachers and decreasing attrition in the profession.

When considering these factors in a larger context, teacher retention is a serious problem that the COVID pandemic has only seemed to exacerbate. Without further knowledge about teachers’ stressors, motivations, and barriers to success that districts and administrators can act on, this crisis will only grow over the coming years. The purpose of the analysis was to better understand factors associated with job dissatisfaction during the COVID-19 pandemic, as measured by self-reported desire to leave the profession, among primary and secondary school teachers in the United States. To accomplish this, we explored a series of individual and system-level factors that may impact retention. We hypothesized that higher levels of dissatisfaction with COVID-19 school policies, prevention strategies, and other job-specific factors would be strongly associated with a desire to leave the profession.

## Materials and methods

Data for this analysis were collected as part of the larger *Monitoring School COVID-19 Mitigation Strategies Project*, funded by the National Foundation for the Centers for Disease Control and Prevention (CDC Foundation) with technical assistance from the CDC, with an aim to better understand COVID-19 prevention strategies in K-12 school environments (CDC Foundation, 2021a,b, c).

### Participants and procedures

As part of this project, a cross-sectional web survey was conducted among educators to collect information on their experiences teaching during the COVID-19 pandemic throughout the 2020–2021 academic years. Qualtrics XM^1^, a global online survey platform, fielded the survey from May 6 to June 8, 2021. Potential respondents were contacted through double-opt-in market research panels, meaning that respondents both volunteered to participate as part of an online panel and confirmed their willingness to participate in a particular survey through an additional email validation process. The number of respondents who clicked into and started the web survey was 6,340. Of those, 4,455 were screened out due to the following screening process checkpoints: (a) did not qualify (e.g., they were not a K-12 teacher), (b) Qualtrics was over quota (e.g., male respondent and the male quota was closed), or (c) respondent stopped at some point in the survey. Thousand eight hundred and eighty five respondents successfully completed the survey. After the survey was completed, Qualtrics performed a data quality check and removed 78 of the 1,885 who completed the survey, due to suspicious bot activity, fraudulent or duplicates, leaving a final sample size of 1,807.

### Research design

The overall design for this investigation was a cross-sectional non-experimental design administered through a one-time survey. Participants were made up of a national-level convenience sample of eligible respondents. Such a design has been successfully implemented by organizations such as the CDC to conduct other national-level surveys on similar topics (Schmidtke and Drinkwater, 2021).

### Sampling

Qualtrics provides access to specific populations through double-opt-in market research panels. Samples are intended to be nationally representative based on U.S. Census estimates and include quotas for demographic characteristics such as gender, race/ethnicity, Census region, and urbanicity. Eligible participants included any full-time K-12 teacher in the United States who was recruited via the survey platform. To increase generalizability, sampling quotas for sociodemographic characteristics (e.g., gender, race/ethnicity, geography) were used based off 2018 data from the National Center for Education Statistics. This study was reviewed and approved by Advarra IRB^2^ a fully accredited independent private Institutional Review Board. Compensation was awarded through points or cash equivalent, which varied depending on their panel arrangement but was estimated to be an average of $10 per completed survey, with a maximum compensation of $15. Compensation was automatically delivered upon quality checks and verification of inclusion.

### Instruments

The questionnaire consisted of 171 items on topics related to respondents’ teaching experiences during the designated timeframe. Questions included self-reported socio-demographics (e.g., age, gender, race/ethnicity) and geographic (urban, rural, suburban) indicators, learning model (in-person, hybrid, virtual), grade(s) taught, COVID-19 specific measures (e.g., COVID-19 status, testing history, in-school COVID-19 mitigation strategies and personal sentiments, vaccine status and sentiments), health/behavioral characteristics (e.g., mental health indicators, coping strategies, social support), and school/district educational support.

### Measures

The primary outcome of interest was a self-reported intention to leave the profession, through retirement or a change in profession altogether. Respondents were asked, “Compared to before the pandemic began (February 2020), how much are you experiencing each of the following?: Thinking about retiring or finding a different profession.” In the three-point Likert-type scale, those stating “more” were classified as being more likely to leave the profession, while those stating either “less,” or “about the same” were classified as not more likely to leave the profession.

### Health/behavioral characteristics

Recent mental health symptoms of depression and anxiety were measured using the Patient Health Questionnaire 2 (PHQ-2) and Generalized Anxiety Disorder (GAD-2) scales, respectively (Bentley et al., 2021). Respondents were asked “Over the last 2 weeks, how often have you been bothered by the following problems?” for the following four items:

• Depression:
  ∘ “Little interest or pleasure in doing things” and
  ∘ “Feeling down, depressed, or hopeless.”
• Anxiety:
  ∘ “Feeling nervous, anxious, or on edge” and
  ∘ “Not being able to stop or control worrying.”

Response options for each item were based on a five-point Likert-type scale using the following scoring method: never (=0), rarely (=1), sometimes (=2), often (=3), and very often (=4). The responses were summed and each respondent was given a score. Respondents were identified as having high, versus low, symptomatology consistent with a particular condition based on pre-determined cutoff values. For both PHQ-2 (depression) (Cronbach α = 0.79) and GAD-2 (anxiety) (Cronbach α = 0.77), composite scores ≥ 3 indicated high symptomatology consistent with these conditions.

Additionally, teachers were asked if they had a health condition that put them at higher risk of serious COVID-19 complications, such as cancer, obesity, asthma, being a smoker, diabetes, heart disease, as well as how likely they thought they were to get exposed to COVID-19 if teaching in-person at school.

### Teacher agency and sentiments characteristics

Respondents reported the degree to which they perceived having personal agency with COVID-19 prevention strategies within their schools and/or districts, as well as satisfaction with those policies. A total of seven questions focused on teachers being given the opportunity to provide input or feedback on district decisions, implementation of COVID-19 prevention strategies (e.g., masks, physical distancing), and their satisfaction with district decisions related to COVID-19 policies and procedures.

How satisfied are you with…

1. School communications about COVID-19 exposures that happen at school.
2. School communications about their plans to open or close due to COVID-19.
3. School decisions to either open or close due to COVID-19.
4. The amount of school supplies offered to students (so they don’t have to share).
5. School decisions requiring students who came in close contact with COVID-19 cases to stay home for a certain amount of time.
6. School decisions allowing students who came in close contact with COVID-19 cases to attend in-person classes without waiting.
7. Amount of COVID-19 supplies for proper hygiene: tissues, hand sanitizer with 60% alcohol, soap, foot pedal trash cans, paper towels, masks.

This series of questions asked about satisfaction with communication and decisions, where response options for each item were based on a five-point Likert scale using the following scoring method: extremely dissatisfied (=1), dissatisfied (=2), neutral (=3), satisfied (=4), and extremely satisfied (=5). The respondent was also able to select not applicable (=0) for each question. NA responses only make up less than 1% of the sample size. We created a satisfaction index score by summing the scores of all seven single items for every participant. The satisfaction index score ranged from 0 to 35. Based on the natural distribution of this index score (left skewed), the data was dichotomized where 25 was used as a cut off. If the score was greater than or equal to 25, then the teacher reported satisfaction with school communications and decisions, otherwise reported dissatisfaction with school communications and decisions (Cronbach α = 0.84).

Similarly, there were a series of questions that asked about the barriers that negatively impacted the school’s ability to implement COVID-19 prevention strategies.

To what extent has each of the following factors negatively impacted your school’s ability to implement COVID-19 mitigation strategies?

1. Lack of funding or resources.
2. Issues with the school’s infrastructure, including availability of hot water, windows in classrooms, and other physical aspects of the school.
3. Lack of technology to support physical distancing in class or remote learning for students and teachers.
4. Lack of personal protective equipment for students, teachers, and support staff, including masks and gloves.
5. Lack of supplies, including cleaning supplies, plastic sheeting, soap or sanitizer, etc.
6. Lack of key staff including teachers, nurses, bus drivers, custodians, and back-up staff.
7. Lack of guidance or collaboration with state and local health departments.
8. Lack of guidance or collaboration with state and local education agencies.
9. Lack of support from the community.
10. Lack of acceptance or adherence to mitigation measures from students or parents.
11. Lack of acceptance or adherence to mitigation measures from teachers or staff.
12. Lack of time to prepare or implement mitigation measures.

Response options for each item were based on a five-point scale using the following scoring method: no impact (=1), little impact (=2), some impact (=3), moderate impact (=4), and significant impact (=5). We created a negative impact of barriers index score by summing the scores of all 12 single items for every participant. The barriers index score ranges from 12 to 60. Based on the distribution of this index score, 36 was used as a cut off. If the score was greater than or equal to 36, then the teacher reported some, moderate or significant negative impact of barriers, otherwise reported little to no negative impact of barriers (Cronbach α = 0.92).

### Data analysis

A multipurpose Iterative Proportional Fitting procedure was used to calibrate individual-level weights, simultaneously adjusting for population estimates from the 2019 National Health Interview Survey; bloc-level non-response adjustment based on calibration in the quintiles of estimated propensity to respond to surveys; and weight trimming procedures (removed 5% of extreme high/low estimates) (Deming and Stephan, 1940; Deville et al., 1993).

A combination of univariate, bivariate, and logistic regression techniques was used to analyze data. We relied on existing literature, knowledge of the dataset, and theoretical models and frameworks to identify a pool of candidate variables for further exploration. From this pool, correlation analyses were conducted to determine a subset of candidate variables associated with the primary outcome of interest while simultaneously addressing multicollinearity concerns. Among these variables, bivariate analyses, using both χ2 and unadjusted odds ratio estimates, were conducted to assess strength of association with the primary outcome. For logistic regression, modeling the best subset selection technique was used to determine which candidate variables offered the highest explanatory value (Affifi et al., 2004). Unless otherwise specified, standard cutoff values of *p* < 0.05 were used to denote significant findings. All analyses were conducted using R version 4.1.0.

## Results

### Characteristics of teachers

Table 1 provides details of relevant variables for respondents overall and by intention to leave, the primary outcome variable. Overall, 43.4% of teachers reported they were thinking about leaving the profession or retiring more so than recalled prior to the COVID-19 pandemic (February 2020). The majority of respondents were White, non-Hispanic, suburban women with household annual incomes of at least $50,000. Results did not vary meaningfully when broken down by intention to leave, with the exception of age. Among all respondents who reported they wanted to leave the profession, a higher proportion of older teachers (≥40 years) stated their increased intention to leave (53.6%) than younger teachers (<40 years) (46.4%).

**TABLE 1:** Characteristics of respondents broken down by teachers’ intention to leave the profession as compared to recalled pre-pandemic intentions to leave, May–June 2021.

	Intention to leave^a^		
	Overall	Less so today or about the same^b^	More so today^b^
Overall N	1,805 (100%)	1,021 (56.6%)	784 (43.4%)
**Sociodemographic characteristics**			
Race/ethnicity			
Non-Hispanic White	1,484 (82.2%)	834 (81.7%)	650 (82.9%)
Non-Hispanic Black	117 (6.5%)	69 (6.8%)	48 (6.1%)
Hispanic	105 (5.8%)	56 (5.5%)	49 (6.3%)
Non-Hispanic Other	99 (5.5%)	62 (6.0%)	37 (4.7%)
Gender			
Female	1,377 (76.3%)	767 (75.1%)	610 (77.8%)
Male	428 (23.7%)	254 (24.9%)	174 (22.2%)
Age			
Under 40 years	947 (52.5%)	583 (57.1%)	364 (46.4%)
40 years or older	858 (47.5%)	438 (42.9%)	420 (53.6%)
Household income			
Under $49,999	283 (15.7%)	175 (17.1%)	108 (13.8%)
Between $50,000 and $99,999	883 (48.9%)	496 (48.6%)	387 (49.3%)
100,000 or more	639 (35.4%)	350 (34.3%)	289 (36.9%)
Geographic location			
Urban	517 (28.6%)	303 (29.7%)	214 (27.3%)
Suburban	903 (50.0%)	515 (50.4%)	388 (49.5%)
Rural	385 (21.4%)	203 (19.9%)	182 (23.2%)
**Health/behavioral characteristics**			
Mental health in past 2 weeks			
Anxiety symptoms^c^	579 (32.1%)	269 (26.4%)	310 (39.5%)
Depressive symptoms^c^	425 (23.6%)	183 (17.9%)	242 (30.9%)
Likelihood to be exposed to COVID-19 if teaching in-person^d^			
Likely	978 (54.2%)	503 (49.3%)	475 (60.6%)
Unlikely or neutral	827 (45.8%)	518 (50.7%)	309 (39.4%)
At higher risk of serious COVID-19 complications^e^			
No or not sure	1,266 (70.1%)	768 (75.2%)	498 (63.5%)
Yes	539 (29.9%)	253 (24.8%)	286 (36.5%)
**Teacher agency and sentiments**			
Given opportunity to provide input or feedback into district decisions about whether to open or close			
No	823 (45.6%)	397 (38.9%*)*	426 (54.4%)
Yes or not sure	982 (54.4%)	624 (61.2%)	358 (45.6%)
Given opportunity to provide input about COVID-19 prevention strategies in the district plan			
No	762 (42.2%)	380 (37.2%)	382 (48.7%)
Yes or not sure	1,043 (57.8%)	641 (62.8%)	402 (51.3%)
Satisfied with district decisions related to COVID-19 policies and procedures			
No	519 (28.8%)	200 (19.6%)	319 (40.7%)
Yes or not sure	1,286 (71.2%)	821 (80.4%)	465 (59.3%)
Satisfaction with school communications, decisions, and supplies related to COVID-19^f^			
Dissatisfied	939 (52.0%)	459 (45.0%)	480 (61.2%)
Satisfied	866 (48.0%)	562 (55.0%)	304 (38.8%)
Impact of barriers on the implementation of COVID-19 prevention strategies			
Little to no negative impact	1,207 (66.9%)	739 (72.4%)	468 (59.7%)
Some, moderate, or significant impact	598 (33.1%)	282 (27.6%)	316 (40.3%)

^a^Please note, that in the top row of the table proportions (Overall N) add to 100% across columns, but within the table itself individual variables (e.g., age, gender) are presented where column percentages add up to 100%.

^b^When referencing to “less so today” or “more so today,” we are measuring teachers’ intention to leave the profession more so at the time of the survey, which was May/June 2021, compared to before the pandemic, which was February 2020.

^c^Using the GAD-2 scale, respondents with anxiety symptoms represent those who scored ≥ 3 for anxiety, indicating a need for follow-up screening for anxiety. Using the PHQ-2 scale, respondents with depression symptoms represent those who scored ≥ 3 for depression, indicating a need for follow-up screening for depression.

^d^Teachers reported how likely they think it is they could get exposed to COVID-19 if teaching in-person.

^e^Question asked teachers if they have a health condition that puts them at higher risk of serious COVID-19 complications (e.g., cancer, obesity, asthma, being a smoker, diabetes, heart disease).

^f^Proportion of respondents who selected “not applicable” to the questions in this index are small (<1%).

With respect to mental health and behavioral characteristics, respondents reporting an increased intention to leave also reported higher levels of anxiety and depression symptoms compared to those without an increased intention to leave. Additionally, a majority (60.6%) of those with increased intention to leave believed they were likely to be exposed to COVID-19 while teaching, compared to 49.3% of those without increased desire to leave. Compared to those without increased intention to leave, a higher proportion of those whose intention to leave increased reported being at high risk for complications from COVID-19 (24.8% vs. 36.5% respectively).

With respect to teacher agency over school policies, higher proportions of those reporting an increased desire to leave also stated they were not given the opportunity to provide input on school opening/closing decisions (54.4%) compared to their counterparts with no increase in intention to leave (38.9%). Additionally, higher proportions of those expressing an increased desire to leave also reported a lack of input on prevention strategies in district plans compared to their counterparts with no increase in intention to leave (48.7% vs. 37.2%). Following this trend, those with an increased desire to leave also reported dissatisfaction with their districts’ overall COVID-19 policies (40.7%), as well as districts’ overall communications, decisions and supplies as it relates to the response (61.2%), compared to their counterparts (19.6 and 45.0% respectively).

### Correlates of increased intentionality to leave the teaching profession

Table 2 presents adjusted and unadjusted odds ratio estimates, and 95% confidence limits, based on teachers’ self-reported intention to leave as recalled prior to the pandemic. Holding all other factors presented in Table 2 constant (e.g., income, gender), teachers aged over 40 years (AOR = 1.87, *p* ≤ 0.001) and reporting household incomes over $100,000 annually (AOR = 1.44, *p* ≤ 0.05) were at greater odds of expressing an increased intention to leave the profession more so than recalled prior to the pandemic than younger teachers or those with household income under $49,000. Teachers reporting symptoms consistent with depression (AOR = 1.50, *p* ≤ 0.01) and anxiety (AOR = 1.35, *p* ≤ 0.01), as measured by the PHQ and GAD scales respectively, were more likely to express an increased intention to leave the profession than those without symptoms. Teachers who reported being at high risk for complications if they caught COVID-19 were at greater odds of increased intention to leave (AOR = 1.44, *p* ≤ 0.01).

**TABLE 2:** Predictors of K-12 teachers thinking about retiring or leaving the profession more so than compared to recalled pre-pandemic intentions: 2020/2021 academic year.

Characteristic	Unadjusted odds ratios (95% CI)	Adjusted odds ratios (95% CI)
**Sociodemographic characteristics**		
Race/ethnicity		
Non-Hispanic White	Referent	Referent
Non-Hispanic Black	0.90 (0.60–1.33)	0.60 (0.32–1.11)
Hispanic	1.09 (0.73–1.63)	1.19 (0.69–2.06)
Non-Hispanic Other	0.77 (0.50–1.19)	0.89 (0.45–1.77)
Gender		
Female	1.15 (0.91–1.44)	0.95 (0.71–1.28)
Male	Referent	Referent
Age		
Under 40 years	Referent	Referent
**40 years or older**	1.55 (1.28–1.88)***	1.87 (1.49 to 2.35)***
Household income		
Under $49,999	Referent	Referent
Between $50,000 and $99,999	1.27 (0.95–1.69)	1.30 (0.94–1.80)
**$100,000 or more**	1.36 (1.01–1.84)*	1.42 (1.00–2.01)*
Geographic location		
Urban	0.95 (0.75–1.19)	0.99 (0.77–1.28)
Suburban	Referent	Referent
Rural	1.16 (0.90–1.48)	1.24 (0.95–1.63)
**Health/behavioral characteristics**		
Mental health in past 2 weeks		
**Depressive symptoms**	1.96 (1.55–2.46)***	1.49 (1.09–2.04)**
Without depressive symptoms	Referent	Referent
**Anxiety symptoms**	1.80 (1.46–2.21)***	1.36 (1.02–1.82)*
Without anxiety symptoms	Referent	Referent
Likelihood to be exposed to COVID-19 if teaching in-person		
**Likely**	**1.59 (1.29–1.96)**	1.22 (0.98–1.52)
Unlikely or neutral	Referent	Referent
At higher risk of serious COVID-19 complications		
No or not sure	Referent	Referent
**Yes**	**1.71 (1.38–2.11)**	1.44 (1.14–1.81)**
**Teacher agency and sentiments**		
Given opportunity to provide input or feedback into district decisions about whether to open or close		
**No**	**1.84 (1.51–2.24)**	1.32 (0.99–1.72)
Yes or not sure	Referent	Referent
Given opportunity to provide input about COVID-19 prevention strategies in the district plan		
**No**	**1.57 (1.29–1.91)**	0.93 (0.71–1.21)
Yes or not sure	Referent	Referent
Satisfied with district decisions related to COVID-19 policies and procedures		
**No**	**2.86 (2.30–3.57)**	1.34 (0.82–2.19)
Yes or not sure	Referent	Referent
Satisfaction with school communications, decisions, and supplies related to COVID-19^a^		
**Dissatisfied**	**1.92 (1.57–2.34)**	1.34 (1.06–1.69)**
Satisfied	Referent	Referent
Negative impact of barriers on the implementation of COVID-19 prevention strategies		
Little to none	Referent	Referent
**Some, moderate, significant**	** (1.49–2.25)**	1.46 (1.15–1.85)**

* < 0.05, ** < 0.01, *** < 0.001.

^a^Proportion of respondents who selected “not applicable” to the questions in this index are small (<1%). Bold values indicates statistically significant findings.

With respect to correlations between increased teacher intention to leave and agency/satisfaction with school policies, those dissatisfied with school communications, decisions, and supplies related to COVID-19 were at 34% greater odds of reporting they were thinking of leaving the profession more than before the pandemic (AOR = 1.34, *p* ≤ 0.01). Additionally, teachers reporting that school district barriers in implementation of COVID-19 prevention strategies had some, moderate, or significant negative impact were at 46% increased odds of increased intention to leave the profession (AOR = 1.46, *p* ≤ 0.01).

To test if district policy sentiments were disproportionately felt across certain groups, Table 3 shows the results of interaction terms. Non-Hispanic Black teachers who reported that they did not have the opportunity to provide input or feedback into district decisions about whether to open or close were at about two and a half times the odds of increased intention to leave compared to their non-Hispanic White counterparts (AOR = 2.43, *p* ≤ 0.05). Additionally, female teachers who reported they were dissatisfied with district decisions around COVID-19 policies and procedures were at 78% increased odds of increased intention to leave compared to their male counterparts (AOR = 1.78, *p* ≤ 0.05).

**TABLE 3:** Interaction terms investigating school policy sentiment by socio-demographic characteristics of K-12 teachers thinking about retiring or leaving the teacher profession more so today than pre-pandemic.

Characteristic	Adjusted odds ratios (95% CI)
**Given opportunity to provide input or feedback into district decisions about whether to open or close**	
No	1.32 (0.99–1.72)
Yes or not sure	Referent
Race/ethnicity	
Non-Hispanic White Non-Hispanic Black	Referent 0.60 (0.32–1.11)
**Hispanic**	1.19 (0.69–2.06)
**Non-Hispanic Other**	0.89 (0.45–1.77)
*Interaction term*: Given opportunity to provide input or feedback into district decisions about whether to open or close * Race/ethnicity	
Teacher Input × Non-Hispanic White	Referent
**Teacher Input ×** **Non-Hispanic Black**	2.43 (1.00–5.91)*
Teacher Input × Hispanic	0.69 (0.26–1.88)
Teacher Input × Non-Hispanic Other	1.06 (0.43–2.58)
**Satisfied with district decisions related to COVID-19 policies and procedures**	
No	1.34 (0.82–2.19)
Yes or not sure	Referent
Gender	
Female	0.95 (0.71–1.28)
Male	Referent
*Interaction term*: Satisfied with district decisions related to COVID-19 policies and procedures * Gender	
**Teacher Satisfaction ×** **Female**	1.78 (1.04–3.04)*
Teacher Satisfaction × Male	Referent

* < 0.05. Bold values indicates statistically significant findings.

### Additional analyses considered

It is important to note that we conducted other analyses to better understand the relationship between teacher intentions and policy sentiment; however, the results were inconclusive. We created two logistic regression models of subsets of the data, in which teachers reported satisfaction versus dissatisfaction with district decisions related to COVID-19 policies and procedures to understand if there was a compounding effect of policy satisfaction with teacher intentions. We also conducted additional mediation analyses where we studied policy satisfaction versus dissatisfaction among the socio-demographic characteristics covered in Table 3, which yielded no new relevant findings. Finally, a multilevel analysis (county-level and individual-level) was conducted to see if two-level multilevel analysis would be a better approach, but county-level variance was around 9% with a confidence interval that crossed zero (0.09, 95% CI: –0.01, 0.19), which could not yield a valid intra cluster coefficient (ICC) estimate, and as a result, did not support altering the analytic approach presented.

## Discussion

The purpose of this analysis was to better understand factors associated with job dissatisfaction among primary and secondary school teachers during the COVID-19 pandemic in the United States (2020–2021 academic year), as measured by a self-reported increased desire to leave the profession. Findings revealed that overall, 43% of K-12 teachers reported a greater desire to leave the profession than they remembered feeling prior to the COVID-19 pandemic (February 2020). We found that increased intention to leave was multi-level, as it was associated with socio-demographic factors (e.g., age, income), individual factors (e.g., self-reported mental health symptoms, perceived COVID risks), as well as teachers’ agency (e.g., dissatisfaction with school/district communications and decisions, as well as perceived barriers to implementation of prevention strategies). We also found demographic disparities with respect to race and gender around both teachers’ ability to provide feedback to schools on opening/closing, and overall dissatisfaction with school/district COVID-19 prevention strategies implementation and policies. Together, these findings are generally consistent with not only the JD-R model, but demonstrate that reasons for leaving are multi-dimensional and reflect an overall dissatisfaction with the way school policies were implemented, the toll the pandemic put on their mental health, and unaddressed safety concerns.

Some findings align with studies from prior to the pandemic that assessed reasons for leaving the profession. For example, in a 2017 Learning Policy Institute research report, 55% of teachers who left the profession reported lack of administrative support, dissatisfaction with teaching as a career, dissatisfaction with working conditions, and dissatisfaction with academic testing and accountability pressures (Carver-Thomas and Darling-Hammond, 2017). Among professional occupations, teachers rate lowest in feeling that their opinions count at work (Greenberg et al., 2016). It is possible that the challenges and barriers teachers reported, such as not being asked to provide input on district COVID-19 policies and procedures and feeling dissatisfied with district decisions related to COVID-19, will exacerbate the already existing challenge of teacher turnover. District leaders continue to have a significant role to play in communication about decisions related to COVID-19. Without teacher input on decision-making, teachers might feel that their perspective is not heard or important. Teachers’ positive perceptions about school leadership (e.g., administrative support, collegial leadership) and colleagues (e.g., teacher connectedness) are associated with lower stress and burnout, as well as higher job satisfaction (Carver-Thomas and Darling-Hammond, 2017; Ouellette et al., 2018; Pressley, 2021). When school leaders, including superintendents and principals, create opportunities for teachers to provide input on decision-making, teachers feel empowered and have higher satisfaction (Mehta et al., 2013; Simon and Johnson, 2015; McConnell, 2017).

Older teachers reported higher odds of increased intention to leave their profession compared to before the pandemic relative to their younger counterparts. Prior studies have demonstrated that desire to leave follows a mostly J-shaped curve, with younger age and older age (vs. mid-career) reporting a greater desire to leave (Carver-Thomas and Darling-Hammond, 2017; Christian-Brandt et al., 2020). In the context of the pandemic, however, other factors known to be associated with occupational change among K-12 teachers, such as having additional non-core responsibilities and having secondary employment may play a greater role (Zimmerman et al., 2020).

We also found that teachers reporting an increased desire to leave also reported symptoms consistent with depression and anxiety, as well as elevated concerns of complications related to COVID-19 exposure. These findings are consistent with research conducted during the pandemic to better understand specific factors related to teacher burnout once in-person teaching resumed in 2020–2021. As Pressley (2021) identified, anxieties about COVID-19, teaching in a new instructional environment, communication with parents, as well as perceived lackluster administrative support, were predictive of K-12 teachers’ burnout-stress in the US (Pressley, 2021). Research from prior to the pandemic also demonstrated the association between stress, burnout and a desire to leave teaching (Christian-Brandt et al., 2020). In 2017, the American Federation of Teachers’ Quality of Life Survey of over 5,000 educators found that 61% of teachers felt work was “always” or “often” stressful (American Federation of Teachers, 2017).

Finally, school policy decisions strongly influenced teachers’ intention to leave. Our study found that dissatisfaction with school communications, and perceived barriers to implementation of policies, were consistently associated with a desire to leave. Additionally, non-Hispanic Black teachers who reported a lack of agency with respect to opening/closing policies had an increased desire to leave, and women teachers dissatisfied with district decisions on COVID policies and procedures had an increased desire to leave compared to before the pandemic. Such findings are consistent with the larger sociological literature on teacher job satisfaction, as they touch on the relationships between autonomy and administrative support, both of which are tied to teacher retention (Klassen and Chiu, 2010; McConnell, 2017). It should be noted that while McConnell (2017) found that high administrative support and teacher autonomy were all strongly correlated with intention of math and science teachers to remain in the profession, the results were not differentiated by gender or race/ethnicity as some were here. While the literature does suggest that gender and racial disparities do exist in feeling autonomy among some teachers, the subject has not been well documented with respect to impacts on retention.

### Limitations

Results should be viewed in light of several limitations. First, data collection occurred at a specific time point during the pandemic (May–June 2021), a complex public health crisis that evolved rapidly and frequently. As such, results may not represent the range of teachers’ experiences across the entire pandemic. Additionally, as this is a one-time, cross-sectional survey, causality cannot be inferred due to temporality bias. Because of this, this study is unable to assess the additional impacts from COVID beyond what is self-reported by respondents. Second, respondent panels are considered non-probability based, convenience samples. Therefore, threats to generalizability remain, albeit buffered by robust sampling quotas and weighting schemes used to increase national representativeness. Third, all results are self-reported, so they are prone to social desirability bias, although research has demonstrated that computer-based surveys are less prone to this effect (Crutzen and Goritz, 2010). Fourth, because school-specific policies and practices exist within a larger political context, potential interactions with school district, community, and state policies may amplify or, conversely, attenuate observed results. Fifth, the disease burden of COVID-19 continues to vary widely by state and by local jurisdictions within states, so the extent to which individuals’ satisfaction with school policies reflected actions commensurate with community disease burden is unknown. It should be noted that a community vulnerability index variable was investigated for its impacts, but ultimately was not predictive and did not make it into the final model. Additionally, there is no way of assessing if levels of dissatisfaction reported here are applicable to other countries, as there is insufficient data to understand the global impacts to the teaching profession. Sixth, responses to the school satisfaction and communications index could impact the results because there were responses that included “not applicable.” There is potential misclassification for those who had NA responses because low index scores can lead to dissatisfaction; however, after reviewing the count, the NA responses only make up less than 1% of the sample size. Seventh, teacher salary would have been a better variable to use as opposed to household income and potentially could be used in further data collection. Household income may not necessarily explain association between income and teachers’ intention to leave. For example, a household income could be more than $300K yet the income of the teacher could be $40K-$50K. Finally, as we are not able to gauge respondents’ familiarity with school decision-making and communication practices, accuracy is assumed without independent verification.

## Implications of work

From a theoretical perspective, the JD-R models suggests pathways between occupational stress and employee burnout that have been empirically replicated in the literature (Hu et al., 2013; Dicke et al., 2018). However, none of this research examining occupational stress through the lens of JD-R has been done in a the context of a global pandemic (Schaufeli and Taris, 2014). In this study, although pandemic-related health concerns were significantly associated with teachers’ increased intentions to leave, the majority of identified influencing factors were organizational and/or came from the education system as the JD-R model predicts. As such, this investigation provides strong support for the model reflecting the relationship between a stressful work environment and job-related outcomes during a health crisis.

From a practical perspective school, district, and community decision-makers all play a role in supporting teacher wellbeing, particularly as it relates to the impact of the COVID-19 pandemic. Despite three federal COVID-19 relief packages, as well as a U.S. Department of Education Return to School Roadmap, both of which were intended to support the academic, physical, and mental health of all members of school communities, it appears that many teachers are still struggling (U.S. Department of Education, 2020, 2021). Teachers’ feeling of lacking satisfactory communication and support during COVID-19 responses and subsequent depressive symptoms can be addressed by school, districts, and community officials, to alleviate teachers’ desire and action to quit, and to prevent academic loss and negative emotions or other educational challenges amongst students.

Additionally, school district leaders can use these findings to support workplace wellness programs, which have shown some success in alleviating workplace stressors and improving retention for teachers (Naghieh et al., 2015). Establishing school health councils or advisory groups that include teachers is also a strategy to consistently gather input and feedback from teachers and staff on decisions related to school health (including emergency preparedness and response) policies and practices that may improve issues of autonomy and support driving job dissatisfaction. Communication between teachers and school administration matters and can be encouraged to reduce gaps in teachers feeling they are not being considered in policy and decision making at schools.

## Conclusion

In conclusion, as the COVID-19 pandemic progresses, demands on teachers are likely to continue as they deal with student learning loss, and stresses on students, families, and community. As specified by the JD-R model, this study demonstrates the need to think about the demands on teachers in a multi-level context. Further research is necessary to understand the resources most useful to teachers in mitigating perceived demands such as input into district- and school-level policies, communication, and implementation. Further, some teachers are more likely than others to leave the field, and educational agencies may wish to target their retention efforts toward these teachers with particular emphasis on employee wellness programs that help educators to manage and reduce their stress, and symptoms of depression and anxiety. Finally, education agency staff may wish to review policies and practices to provide personal support to teachers through strong employee wellness programs.

## Data availability statement

The raw data supporting the conclusions of this article will be made available by the authors, without undue reservation.

## Ethics statement

The studies involving human participants were reviewed and approved by Advarra Institutional Reviews Board. The patients/participants provided their online informed consent to participate in this study.

## Author contributions

AG, RD-D, SL, AW, and LR conceived of the idea and developed the hypothesis. AG performed the computations with assistance from JL. RD-D, JL, and LR verified the analytical methods and overall approach. AG drafted the overall manuscript. KB and LR wrote the introduction. SL and LR reviewed and approved the findings of the work. RG-K and SL wrote the policy implications. AW, SL, and LR reviewed and approved versions of the manuscript. All authors discussed the results and contributed to writing the final manuscript.

## Footnotes

1
https://www.qualtrics.com

2 See 45 C.F.R. part 46; 21 C.F.R. part 56.

## Author disclaimer

**Disclaimer for CDC:** The findings and conclusions in this report are those of the authors and do not necessarily represent the official position of the Centers for Disease Control and Prevention. **Disclaimer for CDCF:** The findings and conclusions in this report are those of the authors and do not necessarily represent the official position of the National Foundation for the Centers for Disease Control and Prevention. This project was funded by the National Foundation for the Centers for Disease Control and Prevention, utilizing funds from its’ COVID-19 Emergency Response Fund. **Disclaimer for Deloitte:** This publication contains general information only and Deloitte is not, by means of this publication, rendering accounting, business, financial, investment, legal, tax, or other professional advice or services. This publication is not a substitute for such professional advice or services, nor should it be used as a basis for any decision or action that may affect your business. Before making any decision or taking any action that may affect your business, you should consult a qualified professional advisor. Deloitte shall not be responsible for any loss sustained by any person who relies on this publication.
